# Evaluation of the In Vitro Synergistic Activity of Ceftazidime/Avibactam Against *Stenotrophomonas maltophilia* Strains in Planktonic and Biofilm Cell Cultures

**DOI:** 10.3390/ph19010001

**Published:** 2025-12-19

**Authors:** Damla Damar-Çelik, Emel Mataraci-Kara, Ayşe İstanbullu-Tosun, Selin Melis Çakmak, Bilge Sümbül, Berna Özbek-Çelik

**Affiliations:** 1 Department of Pharmaceutical Microbiology, Faculty of Pharmacy, Marmara University, 34854 İstanbul, Turkey; damla.damar@marmara.edu.tr; 2 Department of Pharmaceutical Microbiology, Faculty of Pharmacy, Istanbul University, 34116 Istanbul, Turkey; selinmeliscakmak@ogr.iu.edu.tr (S.M.Ç.); berna.ozbek@istanbul.edu.tr (B.Ö.-Ç.); 3 Department of Medical Microbiology, Faculty of Medicine, İstanbul Medipol University, 34214 İstanbul, Turkey; atosun@medipol.edu.tr; 4 Department of Medical Microbiology, Faculty of Medicine, Bezmi Alem University, 34093 İstanbul, Turkey; bsumbul@bezmialem.edu.tr

**Keywords:** ceftazidime/avibactam, synergy, *Stenotrophomonas maltophilia*, biofilm, bactericidal activity

## Abstract

**Background/Objectives**: *Stenotrophomonas maltophilia* (SM) is a significant cause of hospital-acquired infections in immunocompromised and critical care patients. This study investigates the impact of combining ceftazidime/avibactam (CZA) with conventional antibiotics on SM obtained from various sources in planktonic and biofilm cell cultures. **Methods**: Using broth microdilution, the MICs of different antibiotics, including CZA, were determined on 37 SM strains. CZA’s bactericidal and synergistic effectiveness were examined through in vitro time–kill curve tests with tigecycline (TGC), chloramphenicol (CHL), levofloxacin (LVX), colistin (CS), and amikacin (AMK). In addition, synergistic activity was investigated against SM biofilm cell cultures, and antibiotic Mutant Prevention Concentrations (MPCs) were tested against SM isolates. **Results**: Compared to ceftazidime (CAZ), CZA was four times more efficient against 37 SM strains. Unlike TGC and CHL, CS, AMK, and CZA had 2–4 times higher MBCs than MICs. All studied antibiotics were bactericidal at 1× or 4× MIC doses against SM bacteria, except for CZA. The combinations of CZA with LVX and CZA with AMK or CS demonstrated synergistic effects in four out of seven (57%) strains and in three out of seven (43%) strains, respectively, when tested at doses equivalent to the MIC. Moreover, all antibiotic combinations with CZA showed a synergistic effect at dosages four times the MIC. Additionally, CZA and the tested drugs synergistically inhibited SM biofilm formation, and MPC values were 8–16 times the MIC. **Conclusions**: The results of this study indicate that combining CZA with LVX and CS was more effective against SM strains. These combinations might provide alternatives for treating SM pathogens in both planktonic and biofilm cell cultures.

## 1. Introduction

*Stenotrophomonas maltophilia* (SM) is associated with a range of diseases, including pneumonia, bloodstream infections, endocarditis, urinary tract infections, and soft tissue infections. While SM is not a highly virulent pathogen, it has emerged as a significant cause of nosocomial infection associated with crude death rates ranging from 14 to 69% in patients who have bacteremia [1,2,3,4]. SM infections in hospitals are increasing, particularly in patients with compromised immune systems [5,6], and reports have also emerged of community-acquired SM infections [7,8]. Through direct contact with the infection source, susceptible individuals can spread SM to others. Within an intensive care unit, it has been shown that nosocomial SM infections may be transmitted by the hands of medical staff members [9,10]. SM has become a formidable nosocomial pathogen due to its increasing prevalence and capacity for transmission within healthcare settings. Its clinical significance is not solely due to its opportunistic nature, but it is also critically exacerbated by its multidrug resistance profile, which severely restricts effective therapeutic options and contributes to the elevated mortality rates observed in bacteremic patients [11,12].

The high mortality rate among SM patients is partly due to the limited treatment options resulting from multiple antibiotic resistance. SM generates β-lactamases L1 (metallo-beta-lactamase (MBL)) and L2 (cephalosporinase), which confer resistance to penicillins, cephalosporins, and carbapenems [13,14]. Additional resistance mechanisms, including efflux pumps, reductions in antibiotic concentrations within the cells, and antibiotic-inactivating enzymes, are present in these bacteria [1]. These pathways have all contributed to the development of antibiotic resistance in SM. In recent years, resistance to trimethoprim/sulfamethoxazole (TMP-SMX) and levofloxacin (LVX), which are indicated for treating SM infection, has grown [1,15].

Another significant factor in the persistence and treatment resistance of SM is its capacity to build biofilms on both biotic and abiotic surfaces. Biofilm formation facilitates chronic colonization of medical devices such as catheters and ventilators and protects bacterial cells from host immune responses and antibiotic penetration [16]. Nearly all clinical isolates of SM are capable of biofilm production, with key biofilm-associated genes such as *rpfF*, *spgM*, and *rmlA* being highly prevalent. These biofilms significantly complicate treatment, as they reduce antibiotic efficacy and promote multidrug resistance [16,17].

Moreover, biofilms serve as reservoirs for horizontal gene transfer, accelerating the dissemination of resistance determinants such as *sul1*, *sul2*, and *smeDEF* efflux pump genes. This genetic plasticity contributes to bacterial adaptability in hospital environments, where selective pressure from antibiotic use is high. In patients with cystic fibrosis, SM biofilms within the pulmonary tract are associated with chronic colonization and progressive lung function decline, often coexisting with *Pseudomonas aeruginosa* and complicating eradication efforts [18].

Bacteria entrenched in biofilms demonstrate up to 1000 times greater resistance to antibiotics than planktonic cells, often making conventional monotherapies useless. LVX and minocycline are considered to be suitable alternatives, yet their efficacy is variable and strain-dependent [19,20]. In severe cases, combination therapy has been recommended, although robust clinical trial data supporting this approach are lacking. The development of the Infectious Diseases Society of America (IDSA) guidelines from 2022 to 2024 highlights this uncertainty. In 2022, trimethoprim-sulfamethoxazole (TMP-SMX) monotherapy was initially advised as a suitable option; however, in subsequent years, only combination treatment strategies have been recommended [17]. Novel approaches targeting biofilm architecture, including quorum sensing inhibitors, biofilm-disrupting enzymes, and nanoparticle-based delivery systems, are currently under investigation, but they remain experimental [17,21]. The absence of standardized treatment protocols for biofilm-associated SM infections underscores the urgent need to evaluate novel therapeutic strategies [21].

The ceftazidime (CAZ)/avibactam combination drug (CZA) has been developed to combat multidrug-resistant (MDR) Gram-negative bacteria. Additionally, studies have shown that combining CZA and aztreonam has synergistic effects, making it a suitable option for empirical therapy in severe or polymicrobial infections caused by SM [21].

This research aims to evaluate the in vitro susceptibility of CZA, both alone and in combination with other antibiotics, to help control SM infections. The control of these infections is important in clinics because they exhibit molecular heterogeneity related to the distribution of antibiotic resistance and virulence among different strains, as well as a lack of standardized breakpoints for antibiotics that exhibit in vitro activity against this organism [21].

This study examines the bactericidal and synergistic effects of CZA, alone or with colistin (CS), tigecycline (TGC), LVX, amikacin (AMK), and chloramphenicol (CHL), on SM strains in planktonic and biofilm lifestyles. In addition, this study determines the Mutant Prevention Concentrations (MPCs) of the tested antibiotics, which provide information about mutant strain selection forces.

## 2. Results

**Susceptibility testing:** Antibiotic susceptibility testing was performed on 37 SM strains (Supplemental Table S1), and the findings are shown in Table 1. Throughout the study, the antibiotics’ Minimum Inhibitory Concentrations (MICs) we assessed against the American Type Culture Collection (ATCC) strains according to the accuracy range of the Clinical and Laboratory Standards Institute (CLSI) [22]. According to our susceptibility results, CZA showed superior activity against SM strains and exhibited MIC_50_ values four times lower than those of CAZ. TMP-SMX was excluded from the time–kill studies due to its high MIC value. No significant difference was found between the bactericidal and inhibitory endpoints, and the Minimum Bactericidal Concentrations (MBCs) were two to four times the MICs for all drugs except for CHL and TGC.

**Figure 1:**
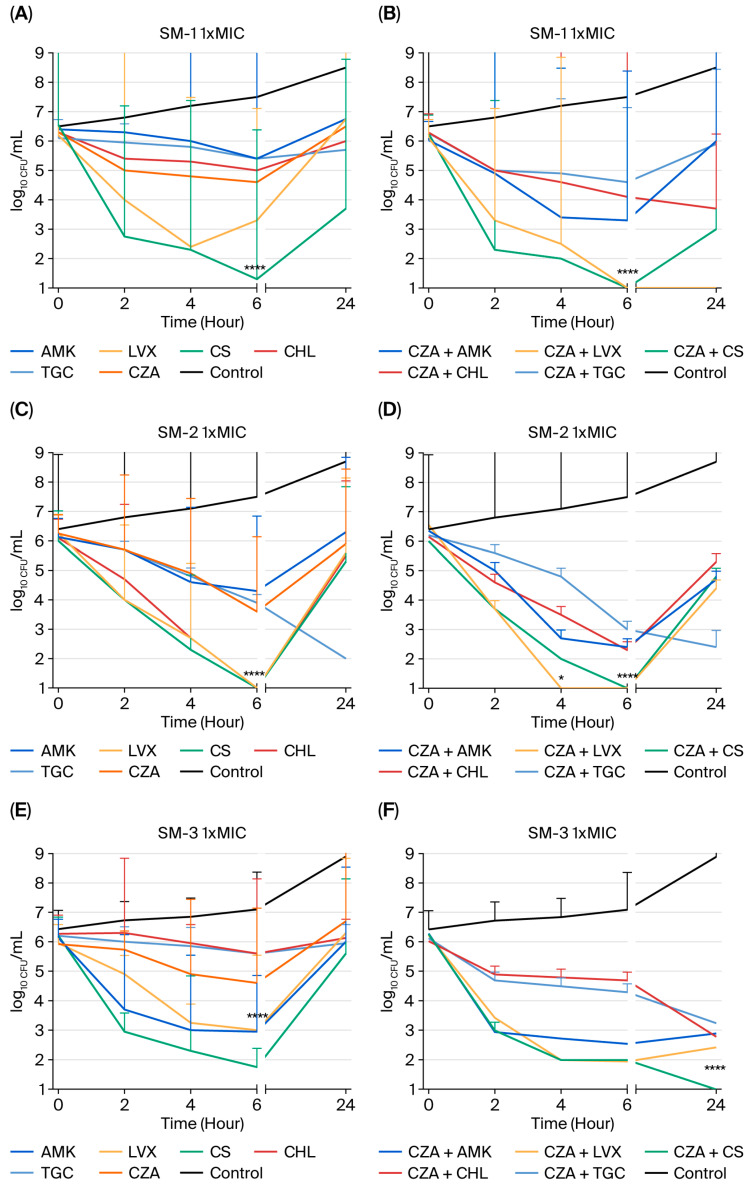

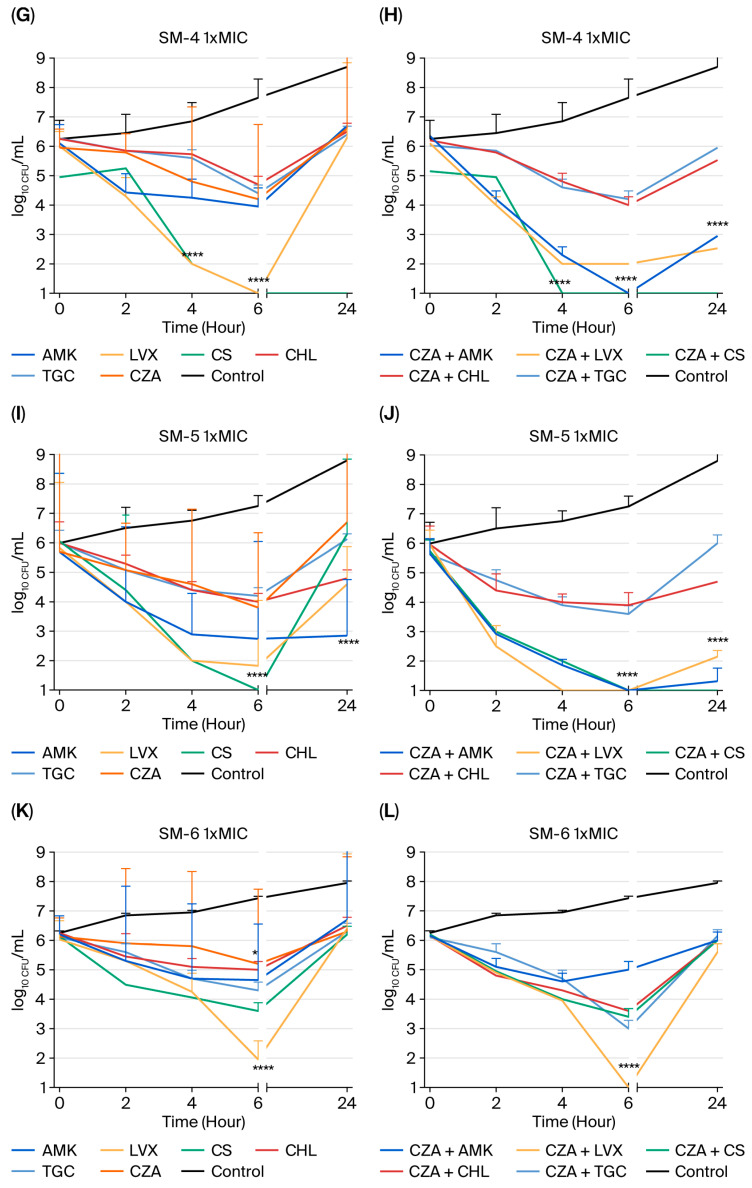

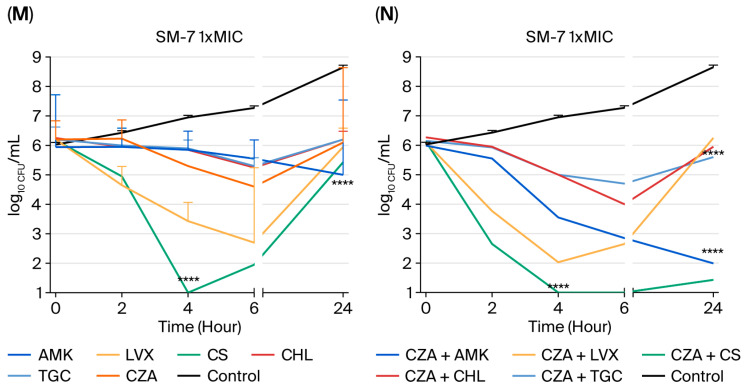
In vitro activities of AMK, LVX, CS, CHL, and TGC alone (1× MIC, mg/L) and in combination with CZA against the SM strains. Time–kill assays were conducted on seven SM strains (SM-1 (**A**,**B**), SM-2 (**C**,**D**), SM-3 (**E**,**F**), SM-4 (**G**,**H**), SM-5 (**I,J**), SM-6 (**K**,**L**), and SM-7 (**M**,**N**)) following exposure to CZA alone (**A**,**C**,**E**,**G**,**I**,**K**,**M**), or in conjunction with amikacin (AMK), levofloxacin (LVX), colistin (CS), chloramphenicol (CHL), or tigecycline (TGC), at concentrations of 1× MIC. The *x*-axis illustrates the duration of killing, while the *y*-axis denotes the logarithmic survival of SM. (* *p* > 0.0001, **** *p* < 0.0001).

**Figure 2:**
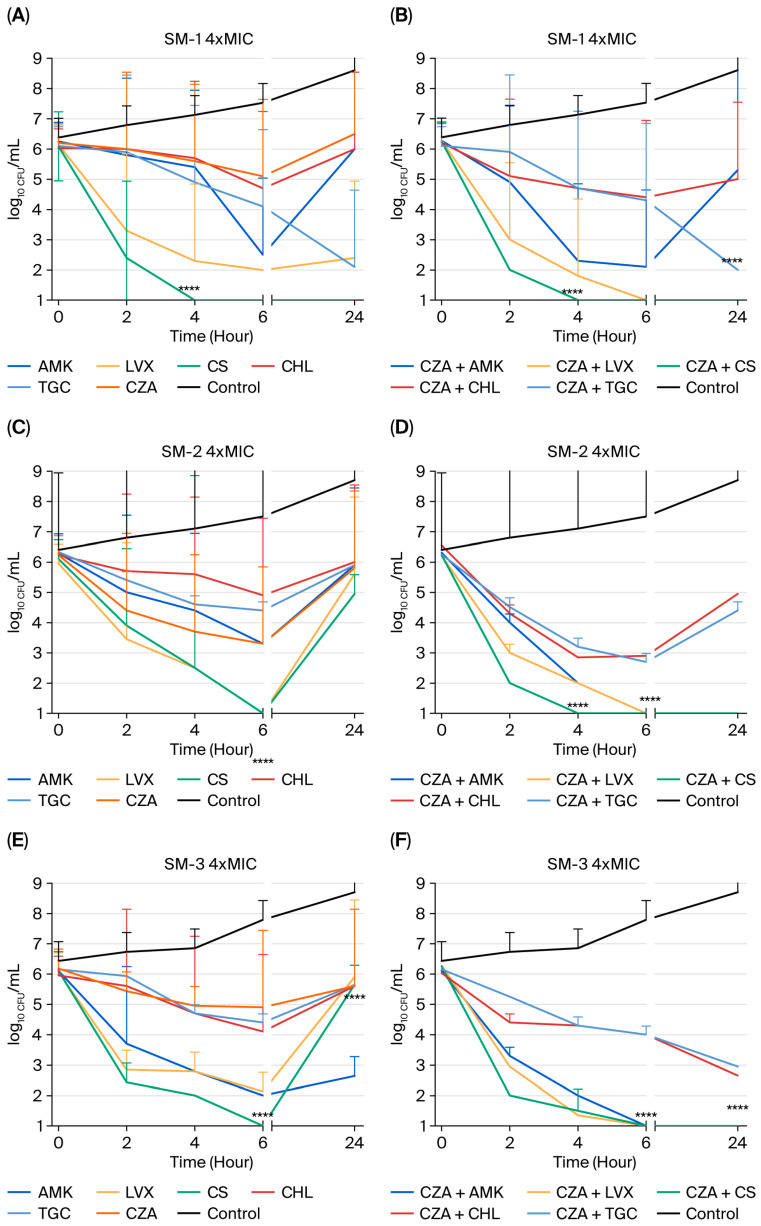

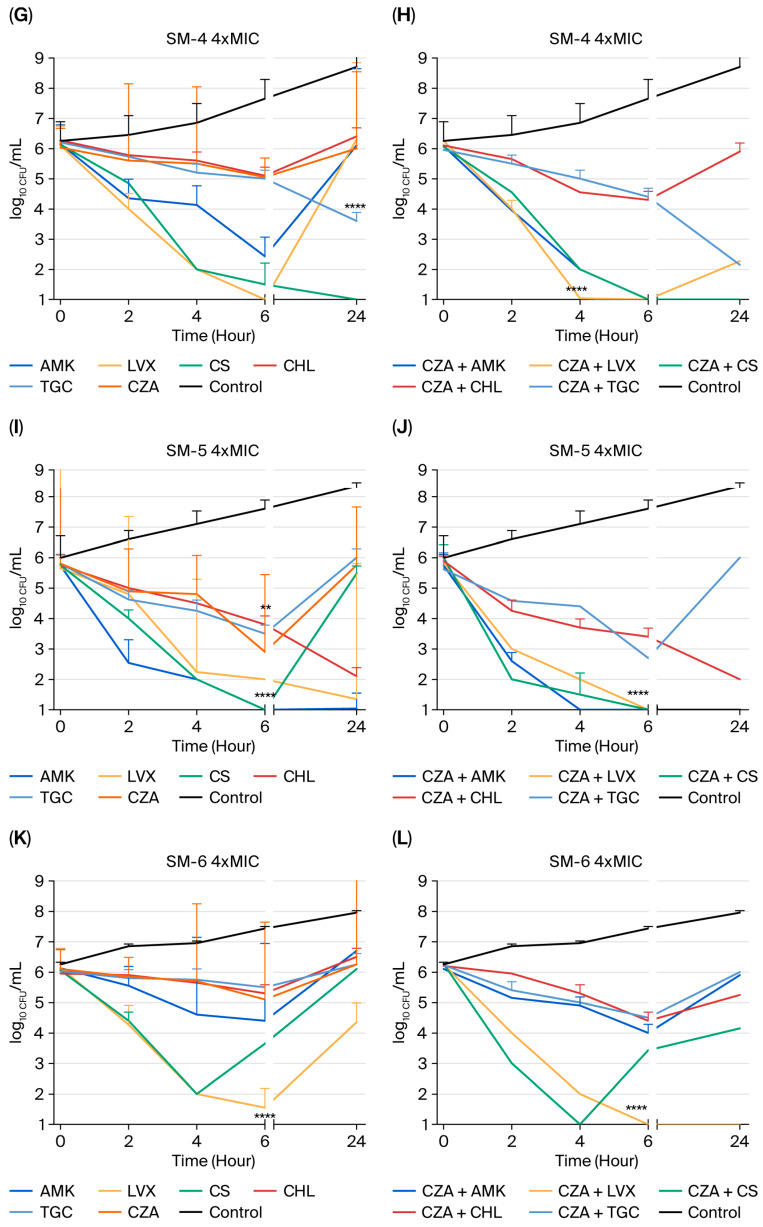

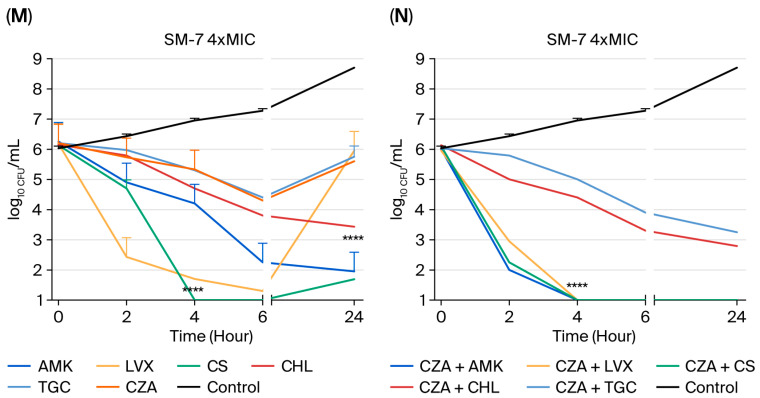
In vitro activities of AMK, LVX, CS, CHL, and TGC alone (4xMIC, mg/L) and in combination with CZA against the SM strains. Time–kill assays were conducted on seven SM strains (SM-1 (**A**,**B**), SM-2 (**C**,**D**), SM-3 (**E**,**F**), SM-4 (**G**,**H**), SM-5 (**I**,**J**), SM-6 (**K**,**L**), and SM-7 (**M**,**N**)) following exposure to CZA alone (**A**,**C**,**E**,**G**,**I**,**K**,**M**), or in conjunction with amikacin (AMK), levofloxacin (LVX), colistin (CS), chloramphenicol (CHL), or tigecycline (TGC), at concentrations of 4× MIC. The x-axis illustrates the duration of killing, while the y-axis denotes the logarithmic survival of SM. (** *p* > 0.0001, **** *p* < 0.0001).

**Time–Kill Study:** Seven isolates (SM-1, SM-2, SM-3, SM-4, SM-5, SM-6, and SM-7) were chosen as representatives, and are described in Table 2. The time–kill curves in Figure 1 consistently show that bactericidal action was achieved within 24 h, resulting in a 3-log_10_ drop in bacterial count, and that all investigated drugs, except CZA, demonstrated bactericidal action against the tested strains at 1× MIC. Furthermore, Figure 2 indicated that bactericidal activity was attained within 24 h, resulting in a 3-log_10_ reduction in bacterial count, and that all examined antibiotics, with the exception of CZA, exhibited bactericidal effects against the tested strains at 4× MIC. While AMK showed a bactericidal effect on SM-5 (Figure 1I) at 1× MIC within 24 h, at 4× MIC, it showed bactericidal activity against three out of seven strains (SM-1 (Figure 2A), SM-5 (Figure 2I), and SM-7 (Figure 2M)). Similarly, LVX exhibited bactericidal activity against SM-1 (Figure 2A) and SM-5 (Figure 2I) at 4× the MIC used. Although CHL did not demonstrate bactericidal activity at 1× MIC, it showed such activity against SM-5 (Figure 2I) when used at 4× MIC. TGC only showed bactericidal activity against SM-2 (Figure 1C) at 1× MIC within 24 h. Moreover, while CS demonstrated bactericidal activity against SM-4 (Figure 1G) at 1× MIC, it showed bactericidal activity against three out of seven strains (SM-1 (Figure 2A), SM-4 (Figure 2G), and SM-7 (Figure 2M)) at 4× MIC within 24 h.

Combining CZA with different antimicrobial agents at 1× MIC resulted in synergistic interactions in several of the strains studied. The most synergistic activity at 1× MIC was found when using the CZA-plus-LVX combination against four out of seven strains (SM-1 (Figure 1B), SM-3 (Figure 1F), SM-4 (Figure 1H), and SM-5 (Figure 1J)), followed by the CZA-plus-CS (SM-3 (Figure 1F), SM-5 (Figure 1J), and SM-7 (Figure 1N)) and CZA-plus-AMK (SM-3 (Figure 1F), SM-4 (Figure 1H), and SM-7 (Figure 1N)) combinations against three out of seven strains. The combinations of CZA with various antibiotics demonstrated synergistic effects against different strains at four times the MIC. The CZA-with-LVX combination showed excellent synergistic activity against all tested strains except for SM-4 (Figure 2H) at 4× MIC within 24 h. In addition, the CZA-plus-CS combination exhibited synergistic activity against five out of seven strains (SM-2 (Figure 2D), SM-3 (Figure 2F), SM-5 (Figure 2J), SM-6 (Figure 2L), and SM-7 (Figure 2N)) at 4× MIC, while CZA plus AMK showed synergistic activity against four out of seven strains (SM-2 (Figure 2D), SM-3 (Figure 2F), SM-4 (Figure 2H), and SM-7 (Figure 2N)) at 4× MIC within 24 h. Figure 1 and Figure 2 show no antagonism in any case [23].

**Estimation of the MPC:** The MPC of CZA for the studied isolate (SM-8) was eight times the MIC of the bacteria. All tested antibiotics’ MPC/MIC ratios are presented in Table 3. The MPC/MIC ratio establishes the concentration range for selecting and amplifying resistant mutant subpopulations. The present study revealed that CZA, LVX, and TGC imposed greater selective pressure for the growth of resistant mutants of the SM strain in comparison to AMK, CS, and CHL, as indicated by the MPC/MIC ratios (Table 3).

**Biofilm-forming ability and Minimum Biofilm Eradication Concentration (MBEC) results:** The biofilm-forming abilities of the 37 tested bacteria are shown in Table 4. The results indicate that most (62%) of the clinical SM isolates, which were isolated from different sources, have strong biofilm-forming capacity, so future studies should investigate the use of this bacterial biofilm cell type in antimicrobial therapy. Furthermore, Table 3 displays the MBEC results. The strain selected for the MBEC analysis was described by Mataraci et al. [24] as showing a strong biofilm-forming ability and relatively lower MIC values in response to the tested antibiotics, as high concentrations of these antibiotics were required for evaluating the MBEC. According to the MBEC results, CZA was ineffective against SM biofilms, similarly to TGC and CHL, and the lowest MBEC value of 64 mg/L was obtained with CS.

**Checkerboard assay results:** Table 5 presents the outcomes of combination trials using the chosen antibiotics in conjunction with CZA against SM-8 biofilms. Synergistic interactions with the SM-8 biofilms were prevalent in almost all combinations, with a fractional inhibitory concentration index (FICI) of ≤0.5 used as the threshold. No antagonism was noted with any combination.

## 3. Discussion

SM is a bacterial species found in various environments that may lead to serious nosocomial infections. The species, formerly susceptible to various agents such as ceftazidime, ticarcillin-clavulanate, trimethoprim/sulfamethoxazole, fluoroquinolones, and tetracyclines, is now showing decreasing susceptibility to these drugs [25]. Monotherapy is often inadequate, especially for immunocompromised patients [3,26]. On the other hand, the high prevalence of biofilm formation among clinical SM isolates significantly contributes to treatment challenges by reducing antibiotic efficacy and promoting multidrug resistance [16,17,18,27]. Therefore, combination treatment is advised due to its synergistic effects and ability to combat resistance [3,26].

CZA might represent a promising new treatment option in these cases, since it combines CAZ, a widely used broad-spectrum anti-pseudomonal cephalosporin, with avibactam, a novel non-β-lactam β-lactamase inhibitor. Avibactam exhibits broad-spectrum activity against various beta-lactamases, including class A (e.g., *ESBLs* and *KPC*), class C (such as *AmpC*), and specific class D enzymes (e.g., *OXA-48*). In February 2015, the FDA allowed CZA in combination with metronidazole to be used for treating complicated urinary tract infections, bacterial pneumonia contracted from hospitals and ventilators, and tough abdominal infections [28,29]. Additional research is needed to examine the synergistic efficacy of combining CZA with other antibiotics in SM infections despite existing evidence of synergistic effects between CZA and aztreonam [21].

In the present study, susceptibility data indicated that CZA was more effective against SM planktonic cells compared to CAZ. However, using CZA alone for SM biofilms was not as effective. On the other hand, an important goals when using the studied antibiotic is preventing selective mutant isolates from entering the population. Our data indicates that CZA showed a significantly lower MPC/MIC value (MPC/MIC = 8, the same as TGC and LVX), compared to CHL, AMK, and CS. Lower values indicate a more remarkable ability of antibiotics to hinder the development of mutants [30,31,32]. Moreover, this study highlighted the synergistic benefits of CZA in combination with AMK, LVX, CS, CHL, and TGC against SM isolates. This interaction indicates that CZA is a promising treatment option for both planktonic and biofilm infections caused by SM [21]. Numerous studies in the literature demonstrate the efficacy of combination therapy in treating SM infections [33,34,35]. For example, Wei et al. showed that SXT + moxifloxacin is more beneficial than monotherapy in inhibiting or killing SM, especially in the case of immunocompromised patients [33].

In addition, Cho et al. showed that TMP-SMX plus ticarcillin–clavulanic acid or LVX exhibited synergistic activity and was more beneficial than monotherapy in inhibiting or killing SM in the bloodstream [34], while Chung et al. found that TMP-SMX plus ticarcillin-clavulanate, CAZ plus AMK, and ticarcillin-clavulanate plus AMK had synergistic effects against trimethoprim-sulfamethoxazole-resistant SM isolates [35].

In the present study, CS had bactericidal effects in one in seven SM strains at 1× MIC (Figure 1G), and in three in seven strains (Figure 2A,G,M) at 4× MIC. At four times the MIC, CZA plus CS synergistically inhibited five in seven SM strains for 24 h (Figure 2D,F,J,L,N), while CZA and CS eliminated three in seven SM strains at all MIC levels. Additionally, CZA with CS synergistically inhibited SM biofilm cells (Table 5). Our analysis found that CS had the lowest MBEC value of 64 mg/L against biofilm cells (Table 3). CS is a last-resort treatment for numerous infections caused by multidrug-resistant Gram-negative bacteria. However, its effectiveness is restricted due to its kidney toxicity; the presence of more advanced, safer antimicrobial drugs; and its inferior results compared to newer commercially accessible agents [36]. Its susceptibility rates in vitro vary from 9% to 86%, and higher doses are usually needed for growth suppression [37,38]. CS combined with other drugs is often studied to combat various resistance mechanisms [36,37,38,39].

In this study, AMK showed bactericidal activity against the studied strains at MIC values of 1× (Figure 1I) and 4× (Figure 2A,I,M) for 24 h. The combination of CZA and AMK exhibited strong synergism against three out of seven (Figure 1F,H,N) and four out of seven (Figure 2D,F,H,N) SM strains, even at the 1× and 4× MIC levels, for 24 h. However, synergistic activity was not exhibited against biofilm cells by either AMK alone or in combination with CZA. AMK has a high MBEC value of 1024 mg/L (Table 3), and the combination of CZA plus AMK was no interaction (Table 5). Due to the important biofilm-forming ability (Table 4) of SM clinical strains, this data could be helpful for assessing treatment options in clinics. SM is inherently resistant to aminoglycosides because of its many aminoglycoside-modifying enzymes and efflux pumps [12,28]. The distribution of these enzymes across SM strains is not consistent, similarly to β-lactamases. Aminoglycosides may be coupled with other antimicrobials to treat severe infections caused by multidrug-resistant (MDR) or extended-drug-resistant (XDR) SM.

Tetracycline derivatives, including minocycline, TGC, and eravacycline, often exhibit low MICs when evaluated against SM in surveillance investigations [40,41,42,43,44]. Tetracycline derivatives are known to quickly spread throughout the body after administration, leading to low levels in the urine and inadequate serum levels [44]. Thus, they are not recommended for treating SM urinary tract infections, and are only approved for combination therapy to treat SM bloodstream infections until blood cultures are clean and clinical improvement is evident [45,46,47]. Building on this pharmacokinetic rationale, the current study evaluated TGC both as monotherapy and in combination with CZA against SM strains. When TGC was utilized alone against SM strains, it showed bactericidal effects (>3 log_10_ killings) in only one strain (Figure 1C) at a 1× dose after 24 h. The combination of CZA and TGC exhibited additive effects against six out of seven and against five out of seven SM strains at 1× and 4× MIC levels, respectively, lasting 24 h. Similarly, this combination exhibited synergistic activity against biofilm cells (Table 5). Moreover, TGC’s MPC value suggests that it is a selectable agent against SM strains, which prevents the selection of mutant strains (Table 3). Thereby, the results of this study suggest that TGC, when combined with CZA, is effective for treating biofilm infections, but alone, it is not effective against biofilm cells under certain conditions.

LVX monotherapy is a suitable treatment for mild SM infections. However, when treating moderate to severe SM infections, it should only be used in conjunction with another active antibiotic. SM isolates often include *Smqnr* resistance genes in their chromosomes that disrupt the binding of fluoroquinolones to gyrase and topoisomerase, resulting in higher fluoroquinolone MICs [48]. Increased development of MDR efflux pumps may further elevate fluoroquinolone MICs [49,50,51]. Pharmacokinetic–pharmacodynamic modeling indicated that fluoroquinolone alone may not effectively reach the desired target levels for treating SM infections, even at large doses. Recent studies show that LVX should be used with care, because it may not be effective when treating SM infections due to its combination of efflux pumps and *Smqnr* genes [52,53,54]. In our study, LVX showed strong bactericidal effects (>3 log_10_ reduction) against two out of seven tested strains (Figure 2A,I) when used at four times the MIC for 24 h. A promising synergistic impact was seen against four out of seven strains (Figure 1B,F,H,J) at 1× MIC, and in six out of seven strains (Figure 2B,D,F,J,L,N) at 4× MIC, when coupled with CZA. Moreover, LVX effectively prevents mutant strain selection, with a value of 4 mg/L MPC, but whether it has a high MBEC for biofilm cells is uncertain (Table 3). However, the combination of CZA and LVX was also found to be synergistic against biofilm cells such as planktonic cells (Table 5).

CHL is the first-line antibiotic recommended by the CLSI [22] for treating SM; however, it is seldom used in the United States because of its adverse effects [55]. In our study, CHL exhibited bactericidal activity against just one out of seven bacterial strains (Figure 2I) at a concentration of 4× MIC. In a prior investigation by Samian et al. [30], the most effective additive or synergistic combinations identified were chloramphenicol with minocycline, ciprofloxacin with imipenem, and ciprofloxacin with meropenem against *Achromobacter xylosoxidans.* Similarly, in the present study, the combination of CZA and CHL had additive impacts against five out of seven strains at 1× MIC, and against six out of seven strains at 4× MIC, after 24 h of usage. Similarly, CHL alone exhibits limited activity in terms of preventing mutant strain selection and eradicating mature biofilms (Table 3). However, when tested in combination with CZA, it was found to be synergistic against biofilm cells (Table 5).

Besides our findings in the present study on combination therapy, we also showed, consistent with the strong biofilm-forming abilities of SM strains described in the literature [16,27], that 23 out of the 37 SM strains from various clinical samples formed strong biofilms (Table 4), highlighting the importance of selecting methods that can also act on biofilms when designing treatment protocols for SM infections.

## 4. Materials and Methods

**Bacterial strains:** For this study, 37 unique SM isolates were chosen at random from a microorganism culture collection maintained at tertiary care centers in Istanbul, Turkey, which included microorganisms previously isolated from patients undergoing hospital treatment; the samples were obtained with appropriate ethical approval. *P. aeruginosa* (ATCC (27853)) and *Escherichia coli* (ATCC (25922)) were used for regular quality control procedures following the guidelines of the CLSI [22]. Mueller–Hinton broth (MHB) from Difco, (Detroit, MI, USA), with 25 mg/L of calcium and 12.5 mg/L of magnesium, was used for the susceptibility tests and time–kill studies (cation-adjusted MHB; CAMHB). Fresh bacterial culture was adjusted in CAMHB at a density of 10^8^ CFU/mL in a McFarland densitometer (Biosan Den-1, Lishui, China) and diluted in an appropriate amount of medium to produce the bacterial inoculum to be used in the experiments. Viable bacterial colonies were counted on tryptic soy agar (TSA; Difco, Detroit, MI, USA) with serial dilution in 0.9% NaCl.

**Antibiotics:** Antimicrobial medications, including CAZ (Sigma Aldrich, St. Louis, MO, USA), TMP-SMX (Sigma Aldrich, St. Louis, MO, USA), CZA (AbbVie Pharmaceuticals, Chicago, IL, USA), avibactam (AVI (Johannesburg, South Africa), final concentration fixed at 4 mg/L), CS (Sanofi Pharmaceuticals, Paris, France), CHL (Thermo Fisher Scientific, Waltham, MA, USA), TGC (Merck, Darmstadt, Germany), AMK (Sigma Aldrich, St. Louis, MO, USA), and LVX (Aventis Pharmaceuticals, Bridgewater, NJ, USA), were supplied by their suppliers. Following the suppliers’ instructions, we produced stock solutions from dry powders and stored them at −80 °C for six months. The TGC solution was freshly made before usage.

**Determining Minimum Inhibitory Concentrations and Minimum Bactericidal Concentrations**: MICs were determined using the broth microdilution technique as specified by the CLSI [22]. MIC values were taken to be the lowest concentration of an antibiotic that halts the observable development of bacteria. Following the incubation period, MBCs were determined by collecting two 0.1 mL samples from wells with no observable growth, and we then cultured them on TSA. After being kept at 37 °C overnight, the colonies were counted. The MBC is the smallest concentration of antibiotics required to eradicate at least 99.9% of the original bacterial population. Each trial was performed three times.

**Time–Kill assay:** The interactions between CZA and AMK, CS, LVX, CHL, and TGC was assessed using time–kill experiments conducted three times in CAMHB with an initial bacterial concentration of 10^6^ CFU/mL. These experiments were performed with CZA combined with each antimicrobial drug at concentrations equal to one and four times the MICs for each bacterium. Seven isolates (SM-1, SM-2, SM-3, SM-4, SM-5, SM-6, and SM-7) with varying susceptibility patterns, as determined by the MIC findings, were subjected to testing using CZA both as a standalone treatment and in combination with other antibiotics. In order to obtain countable colony-forming units (CFUs) for each plate, 0.1 mL samples were obtained from each well at 0, 2, 4, 6, and 24 h. These samples were then diluted with an appropriate proportion of 0.9% NaCl. The samples were plated on TSA agar plates, and the minimum detection limit was 1.0 log_10_ CFU/mL. Time–kill curves were generated by graphing the average colony counts (log_10_ CFU/mL) against time to analyze the 24 h bactericidal effects of single and combination antimicrobial treatments. Each experiment was performed three times.

The results obtained from the time–kill curve analysis were evaluated according to the NCCLS criteria as follows [23]: **Bactericidal activity** is characterized by a more than 3-log_10_ CFU/mL reduction from the starting level. **Synergy** is characterized as a 2-log_10_ CFU/mL increase in lethality after 24 h when the combination is used, in comparison to the most efficacious single therapy. A 2-log_10_ CFU/mL decrease implies **antagonism. The additive effect** had little influence in comparison to the most effective antibiotic [23].

MPCs analysis: A modified version of the method described in references [31,32] was used to identify the MPCs. At 37 °C, one isolate (SM-8) was cultured overnight on TSA medium. Glass tubes were filled with 1 mL of MHB (Difco, Tucker, GA, USA) and then infected with TSA bacteria. The tubes were kept overnight in an incubator at 37 °C with agitation to yield a bacterial concentration of 10^9^ CFU/mL, as assessed counting viable bacterial colonies. To quantify the viable bacterial suspension, it was diluted, and we verified whether the concentration was 10^9^ CFU/mL using an appropriate volume of 0.9% NaCl to ensure that each plate contained countable CFUs. We inoculated 1 mL of each sample onto TSA plates containing tested antibiotics at different doses, from 0.125 to 1024 mg/L, in 2-fold dilutions. After incubation for 48 h, the MPC was determined to be the lowest concentration of antibiotics that completely prevented any observable development of 10^9^ CFU/mL [56]. Each experiment was carried out in triplicate.

**Biofilm-forming abilities:** Thirty-seven strains of SM were evaluated to assess their biofilm-forming capabilities using a 96-well PS microtiter with F-bottom, clear plates (Greiner, Bio-One (Kremsmünster, Austria), Cat. No: 655101), as previously detailed in [24,57,58]. Briefly, two hundred microliters of a crystal violet solution containing 0.1% water was added to every plate, and then the plates were allowed to incubate at room temperature for five minutes. After discoloration was swiftly removed with tap water, the plates were left to air-dry. After the plates had dried, an orbital shaker was used to shake them with 200 µL of 95% ethanol for thirty minutes in order to resolubilize the stain. The optical density at 595 nm (OD 595) was subsequently assessed by using a BioTek EON Microplate Spectrophotometer (Agilent, Santa Clara, CA, USA). The biofilm-forming capacities of the tested isolates were calculated using the method of Stepanovic et al., ranging from non-adherent to strongly adherent [57]. Three distinct tests were performed for each of the 37 SM specimens [24,57].

**Determination of MBEC:** Antimicrobial susceptibility measurements of the biofilm of SM (one isolate- SM-8), known for its robust biofilm-forming capacity, were conducted as previously outlined [24,57] for the MBEC experiments, with the following alterations: Over the course of 24 h, biofilm present on a 96-well tissue culture plate (Greiner, Bio-One, Cat. No: 655101) was washed three times with 250 μL of PBS solution. At CAMHB, antibiotics were diluted in twofold increments, ranging from 1024 mg/L to 0.5 mg/L, and all of these dilutions were produced simultaneously. A 200-microliter aliquot of each concentration was carefully placed into their corresponding wells for a duration of 24 h at 37 °C. Then, the antibiotics were carefully removed from the wells, and the plates were rinsed twice with sterile PBS solution. After the contents were transferred to 1 milliliter of PBS solution, they were subjected to sonication in a Bandelin sonopuls HD 2200 (Bandelin, Berlin, Germany) water bath for 5 min. Subsequently, 100 microliters of each sample was plated on TSA at 37 °C for 24 h. It was determined that the MBEC was the lowest concentration of antibiotics that effectively prevented the multiplication of bacteria. The examinations were conducted thrice.

**Antibacterial activity of antibiotic combinations against biofilms:** The impact of antibiotic combinations on SM-8 biofilms was assessed using the modified broth microdilution checkerboard method [32]. The 24 h biofilms in 96-well tissue culture microtiter plates were rinsed thrice with 250 μL of PBS solution and then air-dried. The activity of the antibiotic combinations was evaluated across six concentrations by measuring the final concentrations in the wells between 16x MIC and MIC/2. Following the incubation of biofilms (initial inoculum in wells at 1 × 10^7^ CFU/mL) with antibiotic combinations at 37 °C for 24 h, the antibiotics were gently aspirated, the plates were washed twice with sterile PBS solution, and the wells were meticulously scraped, with particular attention paid to the well borders. The contents were transferred into 1 mL of PBS solution and subjected to sonication in a water bath for 5 min to disturb the biofilm, after which 100 μL samples were plated on TSA. Colonies were counted during 24 h incubation at 37 °C.

Following the methodology proposed by Mataracı et al. [24], the FICI was determined by dividing the inhibitory concentration of the combination by the inhibitory concentration of the individual antibiotic. The highest dilution of the antibiotic combination that hindered bacterial development was used to calculate the combination index. Synergy was defined as an FICI of less than or equal to 0.5, while no interaction was defined as an FICI ranging from more than 0.5 to 4, and antagonism was defined as an FICI of more than or equal to 4.0.

**Statistical analysis:** All studies were conducted in three separate trials. Two-factor Analysis of Variance with Tukey’s multiple comparison test was used to assess differences between the control group and the groups where antibiotics were administered alone or in combination with ceftazidime/avibactam. While a *p*-value < 0.0001 (****) was considered statistically significant, the others (*) (**) were located at a *p*-value > 0.0001, as shown, and were not statistically significant. 

## 5. Conclusions and Perspectives

In conclusion, the present study highlights that the combination of CZA with LVX, AMK, or CS represents a suitable treatment option for infections caused by SM strains. Furthermore, the superior in vitro MIC_50_ and MIC_90_ values of CZA compared to CAZ make it a superior method for treating SM infections. However, the high biofilm-forming capacity of clinical SM strains should not be overlooked when designing treatment protocols. When considering biofilm formation, it is important to investigate combinations that may also exhibit synergistic effects against biofilms.

On the other hand, this research has some limitations. The sample size of bacterial isolates was small, and the isolates used may not be completely typical of all SM strains. In addition, utilizing 4× MIC of the antibiotics under investigation might have prevented us from attaining the therapeutic levels used to treat SM infections. However, we also achieved a synergistic impact on these isolates at 1× MIC, a concentration achievable in a clinical environment. Even though these tested drugs have the potential to treat SM infections, more studies are needed to explore other resistance mechanisms, such as efflux pumps and porin mutations, in these isolates. Although it is not the main focus of this study, further investigation into resistant isolates’ mechanisms of resistance might be beneficial. However, biofilm studies are difficult and laborious, and we were only able to use a limited number of bacteria from our sample.

Finally, while our study may provide insights into selecting antibiotic combinations for treating SM biofilm infections, further clinical investigations are necessary to evaluate the therapeutic relevance of combining CZA with other drugs and enhancing the effectiveness of this medication against infections caused by SM, especially those associated with a biofilm lifestyle.

## Figures and Tables

**Table 1:** In vitro antimicrobial susceptibility profiles of 37 clinical SM isolates.

Antibiotics	MIC (mg/L)			MBC (mg/L)		
	MIC Range	MIC_50_	MIC_90_	MBC Range	MBC_50_	MBC_90_
CAZ	4–>256	64	256	32–>256	256	>256
CZA *	1/4–128/4	16/4	128/4	8/4–>256/4	64/4	256/4
TGC *	0.5–32	2	16	2–>64	32	>64
LVX	0.25–16	1	4	0.25–256	4	32
CHL	4–>256	8	32	8–>256	128	>256
AMK *	8–>256	64	>256	64–>256	>256	>256
CS *	1–128	4	16	2–>256	16	128
TMP-SMX	1/19–>4/76	4/76	>4/76	>4/76	>4/76	>4/76

The CLSI defines susceptibility and resistance breakpoints for levofloxacin at ≤2 mg/L and ≥8 mg/L, for trimethoprim/sulfamethoxazole at ≤2/38 mg/L and ≥4/76 mg/L, for chloramphenicol at ≤8 mg/L and ≥32 mg/L, and for ceftazidime at ≤8 mg/L and ≥32 mg/L. For the drug mixtures, such as CZA and TMP-SMX, the first number denotes the concentration of the first constituent (CZA or TMP), while the second denotes the concentration of the other constituent (avibactam or sulfamethoxazole). MIC_50_: the antibiotic concentration that inhibits 50% of tested bacterial isolates. MIC_90_: the antibiotic concentration that inhibits 90% of tested bacterial isolates. MBC_50_ and MBC_90_ denote the antibiotic concentrations that kill 50% and 90% of the initial bacterial population, respectively. CAZ: ceftazidime; CZA: ceftazidime/avibactam; TGC: tigecycline; LVX: levofloxacin; CHL: chloramphenicol; AMK: amikacin; CS: colistin; TMP-SMX: trimethoprim/sulfamethoxazole. * The CLSI has not established a susceptibility breakpoint for SM.

**Table 2:** MIC values of the studied strains for the time–kill curve (mg/L).

Antibiotics	Studied Strains in Time–Kill Curve						
	SM-1	SM-2	SM-3	SM-4	SM-5	SM-6	SM-7
CZA	32/4	32/4	64/4	16/4	2/4	2/4	1/4
LVX	4	1	16	0.5	0.5	2	0.25
AMK	16	32	32	64	32	64	16
CS	8	8	2	2	2	0.5	0.5
CHL	16	8	32	8	8	32	16
TGC	16	2	16	4	0.5	2	1

CZA: ceftazidime/avibactam; LVX: levofloxacin; AMK: amikacin; TGC: tigecycline; CHL: chloramphenicol; CS: colistin; MIC: Minimum Inhibitory Concentration.

**Table 3:** MICs, MPCs, and MBECs for different antibiotics against tested SM-8 strain.

Antibiotics		SM-8
Ceftazidime/avibactam	MIC	16
	MPC	128
	MPC/MIC	8
	MBEC	>1024
Colistin	MIC	1
	MPC	16
	MPC/MIC	16
	MBEC	64
Amikacin	MIC	32
	MPC	512
	MPC/MIC	16
	MBEC	1024
Chloramphenicol	MIC	8
	MPC	128
	MPC/MIC	16
	MBEC	>512
Tigecycline	MIC	4
	MPC	32
	MPC/MIC	8
	MBEC	>256
Levofloxacin	MIC	0.5
	MPC	4
	MPC/MIC	8
	MBEC	256

MIC: Minimum Inhibitory Concentration; MPC: Mutant Prevention Concentration; MBEC: Minimum Biofilm Eradication Concentration.

**Table 4:** The distribution of 37 SM strains and their biofilm-forming capabilities.

Biofilm-Forming Ability	Bacteria (Number of Strains%)
Strong adherence	23 (62%)
Moderate adherence	7 (19%)
Weak adherence	5 (13.5%)
No adherence	2 (5.5%)

**Table 5:** In vitro synergy results from chequerboard assays against SM biofilms.

Antibiotic Combination	Synergy	No Interaction
CZA + CS	+	−
CZA + LVX	+	−
CZA + AMK	−	+
CZA + TGC	+	−
CZA + CHL	+	−

FICI stands for fractional inhibitory concentration index. Synergy is characterized by an FIC index of ≤0.5, additivity (absence of interaction) by an FIC value of >0.5–4.0, and antagonism by an FIC index of >4.0. “+” indicates that an effect was seen, while “−” indicates that an effect was not seen.

## Data Availability

The original contributions presented in this study are included in the article/Supplementary Materials. Further inquiries can be directed to the corresponding author.

## Supplementary Materials

The following supporting information can be downloaded at: https://www.mdpi.com/article/10.3390/ph19010001/s1, Table S1: In vitro activities of antibiotics against 37 clinically obtained strains of *S. maltophilia*.

## Abbreviations

The following abbreviations are used in this manuscript:

CZA	ceftazidime/avibactam
SM	*Stenotrophomonas maltophilia*
TMP-SMX	trimethoprim/sulfamethoxazole
MDR	multidrug-resistant
MIC	Minimum Inhibitory Concentration
MPC	Mutant Prevention Concentration
MBEC	Minimum Biofilm Eradication Concentration
AMK	amikacin
LVX	levofloxacin
CS	colistin
CHL	chloramphenicol
TGC	tigecycline
MBC	Minimum Bactericidal Concentration
FICI	fractional inhibitory concentration index
CLSI	Clinical and Laboratory Standards Institute
MHB	Mueller–Hinton broth
CAMHB	cation-adjusted MHB
TSA	tryptic soy agar
CAZ	Ceftazidime
