# Supplementary material for: Evaluation of the In Vitro Synergistic Activity of Ceftazidime/Avibactam Against Stenotrophomonas maltophilia Strains in Planktonic and Biofilm Cell Cultures
Source: Pharmaceuticals (Basel). 2025 Dec 19;19(1):1. doi: 10.3390/ph19010001 (PMC12845402; doi:10.3390/ph19010001)
Supplement: Supplementary file 1 [file pharmaceuticals-19-00001-s001.zip › pharmaceuticals-3911230-supplementary.pdf]

Supplemental Table S1: In vitro activities of antibiotics against 37 clinically obtained strains of *S.maltophilia*.

	**CZA***	**CAZ**	**TGC**	**CHL**	**LVX**	**TMP-SMX**	**AMK**	**CS**
**SM-1**	32	64	16	16	4	20/380	16	8
**SM-2**	32	64	2	8	1	>40/760	32	8
**SM-3**	64	128	16	32	16	40/760	32	2
**SM-4**	16	32	4	8	0.5	40/760	64	2
**SM-5**	2	8	0.5	8	0.5	1.25/23.75	32	2
**SM-6**	2	32	2	32	2	20/380	64	0.5
**SM-7**	1	8	1	16	0.25	40/760	16	0.5
**SM-8**	16	64	4	8	0.5	40/760	32	1
SM-9	256	>512	4	32	8	>40/760	>512	2
SM-10	64	256	4	8	1	>40/760	256	16
SM-11	32	256	1	4	1	>40/760	16	2
SM-12	2	8	4	8	2	20/380	64	2
SM-13	256	>512	2	16	4	20/380	128	4
SM-14	128	256	4	8	1	>40/760	256	128
SM-15	4	8	0.5	8	0.5	20/380	64	2
SM-16	32	64	2	8	0.5	>40/760	64	1
SM-17	2	16	4	16	1	>40/760	256	8
SM-18	4	8	1	8	0.5	20/380	64	2
SM-19	16	64	1	4	0.25	20/380	64	1
SM-20	64	128	8	16	2	20/380	>512	1
SM-21	16	64	2	512	1	>40/760	>512	8
SM-22	64	256	32	16	0.5	>40/760	256	4
SM-23	8	32	1	16	0.25	10/190	64	8
SM-24	4	8	4	32	0.5	20/380	128	1
SM-25	64	256	2	8	1	10/190	256	64
SM-26	128	256	2	16	1	40/760	64	8
SM-27	8	128	16	8	2	1/19	>512	64
SM-28	1	4	1	4	0.25	10/190	32	4
SM-29	32	128	8	4	0.5	10/190	256	8
SM-30	16	32	1	4	0.25	20/380	256	8
SM-31	16	64	32	128	16	>40/760	>512	256
SM-32	64	256	2	8	2	>40/760	256	8
SM-33	128	256	1	8	1	10/190	256	4
SM-34	1	4	8	8	0.25	10/190	32	0.25
SM-35	2	4	1	4	0.5	20/380	32	4
SM-36	16	32	2	32	1	40/760	>512	1
SM-37	16	64	16	64	8	>40/760	>512	16

* Avibactam (AVI) final concentration fixed at 4mg/L.

CLSI defines susceptibility and resistance breakpoints for levofloxacin at ≤ 2 mg/L and ≥ 8 mg/L, for trimethoprim/sulfametaxazole at ≤ 2/38 mg/L and ≥ 4/76 mg/L, for chloramphenicol at ≤ 8 mg/L and ≥32 mg/L, for ceftazidime at ≤ 8 mg/L and ≥32 mg/L. For the mixture drug, such as

TMP-SMX, the first number denotes concentration of the first constituent (TMP), while the other denotes concentration of the other constituent (sulfametohoxasole).

CAZ: ceftazidime; CZA: ceftazidime/avibactam; TGC: tigecycline; LVX: levofloxacin; CHL: chloramphenicol; AMK: amikacin; CS: colistin; TMP-SMX: trimethoprim/sulfametaxazole.
